# Global Health in Preconception, Pregnancy and Postpartum Alliance: development of an international consumer and community involvement framework

**DOI:** 10.1186/s40900-020-00218-1

**Published:** 2020-08-10

**Authors:** Heidi J. Bergmeier, Virginia Vandall-Walker, Magdalena Skrybant, Helena J. Teede, Cate Bailey, Jo-Anna B. Baxter, Ana Luiza Vilela Borges, Jacqueline A. Boyle, Ayesha Everitt, Cheryce L. Harrison, Margely Herrera, Briony Hill, Brian Jack, Samuel Jones, Laura Jorgensen, Siew Lim, Cynthia Montanaro, Leanne M. Redman, Judith Stephenson, Hildrun Sundseth, Shakila Thangaratinam, Paula Thynne, Ruth Walker, Helen Skouteris

**Affiliations:** 1 Monash Centre for Health Research and Implementation, School of Public Health and Preventive Medicine, Monash University, Clayton, Victoria Australia; 2 Faculty of Health Disciplines, Athabasca University, Athabasca, Canada; 3 NIHR Applied Reseach Collaboration West Midlands, Midlands, UK; 4 Institute of Applied Health Research, University of Birmingham, Edgbaston, Birmingham, UK; 5 Monash Partners Advanced Health Research Translation Centre, Clayton, Victoria Australia; 6 Monash Health, Melbourne, Clayton, Victoria Australia; 7 Centre for Global Child Health, The Hospital for Sick Children, Peter Gilgan Centre for Research and Learning, Toronto, ON Canada; 8 Public Health Nursing Department, University of Sao Paulo, Sao Paulo, Brazil; 9 HiPPP Consumer Expert Group, Monash Centre for Health Research and Implementation, School of Public Health and Preventive Medicine, Monash University, Clayton, Victoria Australia; 10 Boston University Institute for Health Systems Innovation and Policy, Boston, MA USA; 11 Barts Research Centre for Women’s Health (BARC), Women’s Health Research Unit, Centre for Primary Care and Public Health, Blizard Institute, Barts and The London School of Medicine and Dentistry, London, UK; 12 Wellington-Dufferin-Guelph Public Health, 160 Chancellors Way, Guelph, Ontario Canada; 13 Reproductive Endocrinology and Women’s Health Laboratory, Pennington Biomedical Research Center, Baton Rouge, USA; 14 Institute of Women’s Health, University College London, EGA Institute for Women’s Health, London, UK; 15 European Institute of Women’s Health, Dublin, Republic of Ireland; 16 Institute of Metabolism and Systems Research, WHO Collaborating Centre for Women’s Health University of Birmingham, Birmingham, UK; 17 Warwick Business School, University of Warwick, Coventry, UK

**Keywords:** Preconception, Pregnancy, Postpartum, Obesity, Consumer and community involvement, Framework, Patient and public involvement, Stakeholder engagement

## Abstract

**Background:**

The goal of the Global Health in Preconception, Pregnancy and Postpartum (HiPPP) Alliance, comprising consumers and leading international multidisciplinary academics and clinicians, is to generate research and translation priorities and build international collaboration around healthy lifestyle and obesity prevention among women across the reproductive years. In doing so, we actively seek to involve consumers in research, implementation and translation initiatives. There are limited frameworks specifically designed to involve women across the key obesity prevention windows before (preconception), during and after pregnancy (postpartum). The aim of this paper is to outline our strategy for the development of the HiPPP Consumer and Community (CCI) Framework, with consumers as central to co-designed, co-implemented and co-disseminated research and translation.

**Method:**

The development of the framework involved three phases: In Phase 1, 21 Global HiPPP Alliance members participated in a CCI workshop to propose and discuss values and approaches for framework development; Phase 2 comprised a search of peer-reviewed and grey literature for existing CCI frameworks and resources; and Phase 3 entailed collaboration with consumers (i.e., members of the public with lived experience of weight/lifestyle issues in preconception, pregnancy and postpartum) and international CCI experts to workshop and refine the HiPPP CCI Framework (guided by Phases 1 and 2).

**Results:**

The HiPPP CCI Framework’s values and approaches identified in Phases 1–2 and further refined in Phase 3 were summarized under the following five key principles: 1. Inclusive, 2. Flexible, 3. Transparent, 4. Equitable, and 5. Adaptable. The HiPPP Framework describes values and approaches for involving consumers in research initiatives from design to translation that focus on improving healthy lifestyles and preventing obesity specifically before, during and after pregnancy; importantly it takes into consideration common barriers to partnering in obesity research during perinatal life stages, such as limited availability associated with family caregiving responsibilities.

**Conclusion:**

The HiPPP CCI Framework aims to describe approaches for implementing meaningful CCI initiatives with women in preconception, pregnancy and postpartum periods. Evaluation of the framework is now needed to understand how effective it is in facilitating meaningful involvement for consumers, researchers and clinicians, and its impact on research to improve healthy lifestyle outcomes.

## Plain English summary

Obesity is a major risk factor for developing serious chronic health conditions. Younger women between the ages of 20 and 40 years are gaining weight more rapidly than men and women in other age ranges. Excess body weight before and during pregnancy can have a severe negative impact on mothers and babies, as well as on their future health. While excess body weight after birth has been linked with women’s long-term weight retention, further weight gain and unhealthy lifestyle behaviours that increase the risk of obesity for mothers and their children. As such, there is global agreement of the urgent need to address maternal obesity.

Research shows that healthy lifestyle interventions (that focus on improving healthy eating and physical activity behaviours) before, during and after pregnancy, are effective in reducing excess weight gain. However, it is not yet clear how this research evidence can reach and be applied by clinicians and families in the real world.

We formed the Health in Preconception, Pregnancy and Postpartum (HIPPP) Global Alliance to address this problem. The Alliance comprises consumers (members of the public), clinicians and practitioners from five continents. Our goal is to produce research and translation priorities around healthy lifestyle and obesity prevention with women across their reproductive years. New knowledge cannot be produced and translated successfully without thorough consumer involvement. In this paper, we outline our strategy for the development of a framework that will guide how consumers will be involved in each stage of our research and translation processes.

## Introduction

There is global consensus to urgently address maternal obesity [1, 2]. In the setting of increasing weight at a global population level, women between the reproductive ages of 20–40 years represent the population group at highest risk of obesity development [3]. Increased weight prior to pregnancy, excess gestational weight gain and postpartum weight retention are significant and independent contributors to rising maternal obesity [4–6]. Maternal obesity is evident across low, middle and high-income countries [7], however obesity prevalence is growing faster among populations experiencing socio-economic challenges [8, 9]. Maternal obesity is associated with adverse health outcomes in pregnancy and increased risk of chronic disease in women, including coronary heart disease, stroke, type 2 diabetes, as well as asthma, poorer cognition, neurodevelopmental disorders and obesity development in the child [3, 4, 10]. Long-term lifestyle and pharmacological treatment of obesity are largely ineffective and have not curbed associated adverse health outcomes [11, 12].

The Health in Preconception, Pregnancy and Postpartum (HiPPP) Global Alliance (the Alliance) was formed in 2018 in response to the impact of overweight and obesity among women before, during and after pregnancy [11, 13]. The Alliance comprises international, multidisciplinary, expert stakeholders across consumer, community, government, private and public health services, workplaces and primary care, representing leading international academic bodies [11, 13, 14]. Our agreed aims include: 1) to establish priorities for preconception, including inter-conception (the time between pregnancies), pregnancy and postpartum healthy lifestyle and care to prevent maternal obesity and related pregnancy and long-term complications (priorities were developed using a modified Delphi and Nominal Group Technique) [14]; 2) to review quality, identify gaps and update evidence-based guidelines for weight and lifestyle management in HiPPP; 3) to co-develop workforce development priorities and strategies; 4) to establish a HiPPP international early- and mid-career network; and 5) to co-develop consumer involvement priorities for HiPPP and maternal obesity prevention [11, 13]. A consumer (also referred to as a member of the public, patient and stakeholder in other countries) is historically the term used widely throughout Australia as reflected in the title of the National Health and Medical Research Council’s Statement on Consumer and Community Involvement (2016) [15] and has become the term that people relate to in Australia to describe any person affected by the research, such as those with a lived experience of a health condition and/or a recipient of research knowledge/health and community services [15, 16]. There was consensus that the priorities for HiPPP and maternal obesity prevention would be co-developed with consumers. In this paper we focus on the development of the HiPPP Consumer and Community Involvement (CCI) Framework to ensure that people who will be affected by and/or who may benefit from the research have opportunity to be involved meaningfully throughout the process.

Research by our group and others demonstrates that low-intensity lifestyle interventions in reproductive aged women, including in preconception, pregnancy and postpartum, prevents weight gain [17–19]. Yet without implementation research to drive evidence into practice, this knowledge remains siloed, failing in scale up and delivery of health impact. New knowledge is vital about “how” to implement systems-wide, evidence-based strategies. This new knowledge cannot be generated and translated effectively without rigorous consumer and community involvement to ensure interventions are relevant and feasible for those using them [13]. However, significant barriers impede women participating in, and partnering with researchers, during key obesity prevention windows in preconception, pregnancy and postpartum life stages [20, 21]. These barriers include: 1) the transient nature of perinatal life stages with limited timeframes; 2) the normative nature of perinatal life stages, where women are not viewed as “patients” and not always aware of opportunities to be involved in health and medical research; and 3) the challenges of dealing with a new baby which limit self care and availability. To address these challenges, the HiPPP Alliance prioritised the development of a CCI framework designed to overcome some of these barriers and to enable consumer participation and partnerships so as to establish not only what “what works” but also “what translates” into meaningful health outcomes for women and the next generation.

The involvement of consumers and community in health research is not new. Consumer and community involvement has increased significantly over the past decade since being introduced in the United Kingdom (UK) 20 years ago [22], and is now a policy directive in countries including Australia [15], the UK [22], Canada [23], and the United States [24]. These countries apply different nomenclature such as patient engagement (US) and patient-researcher engagement (CA), patient and public involvement (UK) or consumer and community involvement (AUS). The aim of CCI is to improve the quality, direction, relevance and impact of health and medical research as well as help to prioritise resources and activities [24–26].

Consumer and community involvement in research refers to the active partnership between researchers and those affected by the research, in contrast to having research conducted for them [15, 16, 25, 27]. As well as consumers, CCI may include relevant communities, such as organisations, services and settings (e.g., community health centres, hospitals, cultural groups) that may play a role in informing the solution. In this paper, we focus on the work conducted with researchers and consumers. Consumer and community involvement can take many forms during all stages of research and translation, from that of participant to partner [16]. As research partners, consumers can be involved across the research continuum from identifying the problem, priority setting, attracting funding to governance, research co-design, interpretation of data and publication and presentation of the findings [15, 25]. In line with the growing acceptance of the imperative to embed CCI in research and translation initiatives, recent efforts have focused on: 1) conceptualising CCI [28]; 2) identifying the guiding principles needed for successful CCI and researcher interactions [29]; and 3) evaluating the impact of CCI [30]. More recently, the UK National Institute for Health Research (NIHR) reviewed CCI (referred to as Patient and Public Involvement in the UK) progress over the past decade [22]. This work highlighted that despite meaningful advances, including increases in infrastructure to support CCI, there is still a need for the field to mature from ‘tokenism’ to meaningful involvement of consumers and researchers. A key finding was a lack of consistent reporting of CCI in research initiatives, limiting our understanding of “what works” and preventing translation of “what we know” about CCI in research and translation to “what we do” [31].

In this paper we respond to the NIHR review [22] recommendation by reporting on the development of the international HiPPP CCI Framework. Given the need to adapt local contexts and the evolving nature of the field, we focus on progress to date and acknowledge that this work will continue to evolve in response to the needs of consumers, researchers, health professionals and other stakeholders as well as the HiPPP objectives and opportunities.

## Methods and results

**Fig. 1:**
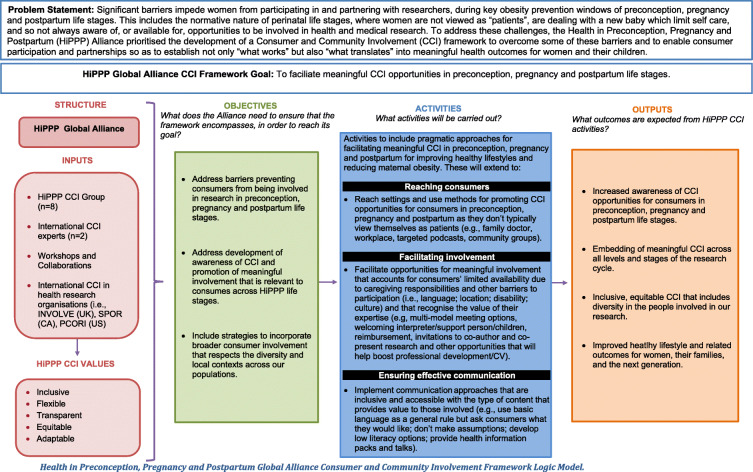
Health in Preconception, Pregnancy and Postpartum Global Alliance Consumer and Community Involvement Framework Logic Model

In accordance with the development of other CCI frameworks [32], the HiPPP CCI Framework was developed using a pragmatic mixed method iterative process with a combination of workshops (involving researchers and consumers), a literature review, and qualitative research. This three-phase process is summarised in Table 1 and outlined below. The logic model shown in Fig. 1 provides a graphic representation that describes how the framework is proposed to work. It articulates the ways that the CCI team can undertake their part of the framework implementation, including the values and approaches that need to be operationalised. To contribute toward the improvement of the international CCI knowledge base, we report this work using the GRIPP2 (Guidance for Reporting Involvement of Patients and the Public) Short Form [33] (Table 2).

**Table 1:** HiPPP Consumer and Community Involvement Framework Co-Development Process

Phases	Values	Implementation
**Phase 1**	**Inclusive**	
CCI Workshop, with 13 international experts in the fields of women’s preconception, pregnancy and postpartum health, two consumers and six early career researchers representing five continents, to propose and discuss values and approaches for the framework development.	HiPPP: • Works with consumers to understand what is needed to ensure that consumer involvement opportunities are inclusive for women with lived experience of weight/healthy lifestyle issues, regardless of their background, education, location, age, culture and language. This includes holding meetings in appropriate venues (i.e., child friendly), providing interpreters, welcoming support people (i.e., partner) and adapting processes for cultural relevance. • Has mechanisms and processes to ensure that consumers are compensated for their involvement. This may include reimbursement of transport and childcare costs. • Produces Plain english summaries of research available for consumers and local communities.	HiPPP implements pragmatic approaches for facilitating meaningful CCI in preconception, pregnancy and postpartum for improving healthy lifestyle and reducing maternal obesity; that extend to:
		**Reaching consumers**
		HiPPP reaches settings and uses methods for promoting CCI opportunities for women in preconception, pregnancy and postpartum as they don’t typically view themselves as patients: • Family doctor/general practitioner • Hospitals • Specialist care (fertility clinics; endocrinologists) • Waiting rooms of healthcare providers • Workplace • Community centres • Targeted podcasts • Targeted mobile apps • Retail outlets (e.g., charity shops) • CCI registration database • Pharmacy • Playgroups • Library (including toy library) • Community groups and centres (including cultural/language group) • Support services/programs for young mothers • Parent rooms in shopping centres, workplaces
**Phase 2**	**Flexible**	
Review of peer-reviewed and grey literature to identify existing CCI frameworks and resources.	HiPPP: • Partners with consumers and communities to identify different ways they can be involved in research projects at different stages of the research cycle, that take into account flexible options required to facilitate meaningful involvement during preconception, pregnancy and postpartum. • Makes decisions on locations, frequency, timing of meetings with consumers and local communities. This includes making sure that opportunities for involvement are held at times and places that best suit women/relevant consumers at different stages of preconception, pregnancy and postpartum. • Uses a variety of methods to involve consumers and local communities targeted at specific stages of preconception, pregnancy and postpartum or as a continuum. This includes a CCI section on HiPPP webpages, social media platforms (e.g. Facebook, Whatsapp) and promotion through poster/leaflet campaigns at playgroups, workforces and hospitals.	
**Phase 3**		
Building on findings from *Phases 1* and *2*, collaboration with consumers (i.e., members of the public with lived experience of weight/lifestyle issues in preconception, pregnancy and postpartum) and international CCI experts to workshop and refine the framework.		
	**Transparent**	
	HiPPP: • Presents opportunities to be involved in research projects in a clear, accessible format and will include information about expectations for the role, expected time commitment, information about reimbursement and contact details for the lead researcher. • Advertises opportunities for involvement in a range of places, including: community centres, hospitals, support groups, playgroups and the workforce. • Is clear about who needs to be recruited and why people have been chosen to be involved (e.g. relevant experience) when opportunities for involvement are advertised.	
		**Facilitating involvement**
		HiPPP facilitates opportunities for meaningful involvement that account for consumers’ limited availability due to caregiving responsibilities and other barriers to participation (i.e., language; location; disability; culture) and recognises the value of the expertise that they contribute; approaches include: • Online, telephone and face-to-face meetings. • Reimbursement for travel/ childcare costs. • Welcoming a support friend/relative at meetings. • Welcoming/providing an interpreter. • Meeting in child-friendly venues (i.e., play groups; child activities where parents are sitting around waiting). • Providing afternoon tea or equivalent. • Providing preconception starter kit with evidence-based advice and source of information clearly identified and referenced (other online and advice not always accurate/consistent). • Offering vouchers for education sessions (e.g., consultation with pre-pregnancy dietitian)/information sessions. • Invitations to co-author and co-present research and other opportunities that will help boost professional development/CV. • Providing certificates to recognise training received or involvement.
	**Equitable**	
	HiPPP: • Views researcher-consumer relationships based on mutual trust, integrity and respect as central to implementing meaningful CCI. • Recognises the value that both the researcher and consumer contribute toward research. • Involves consumers in designing and agreeing on CCI strategies. • Ensures that researchers working with consumers have the skills to facilitate appropriate group processes, such as fostering a culture that views researchers and consumers as equal partners, creates opportunities for speaking and listening and welcomes diverse viewpoints. • Partners with consumers to identify ways to publicly acknowledge the value of their contribution, such as co-authoring publications and co-presenting at conferences. • Recognises that both consumers and researchers may require training and support for implementing CCI. • Avoids making assumptions about consumers knowledge and capacity to learn, and partners with them to understand their preferences around the use of medical terminology.	
		**Effective communication**
		HiPPP implements communication approaches that are inclusive and accessible and the type of content that provides value to those involved, including: • Using basic English as a general rule but asking consumers what they would like (don’t make assumptions). • Providing options for low literacy levels. • Through Maternal Child Health Nurse information packs. • As short brief messages. • Through social media posts and videos. • Providing health updates and tips. • Providing informative talks to families (mothers and fathers).
	**Adaptable**	
	HiPPP: • Evaluates researcher and consumer experiences and involvement at set intervals throughout the project and adapts processes as the needs of consumers, researchers and project change. • Nurtures a culture where consumers feel comfortable providing feedback about their experiences and discuss altering their roles at any stage. • Ensures that consumers have a clear idea of how their involvement contributes toward the research and its outcomes. • Develops methods to evaluate the impact of CCI on improving healthy lifestyle of women in preconception, pregnancy and postpartum. • Provides regular feedback to consumers and communities on their involvement in projects and in turn receives feedback on consumers’ reflections on experiences of involvement, making changes to involvement approaches where necessary.	

*CCI=Consumer and Community Involvement; HiPPP=Health in Preconception, Pregnancy and Postpartum*

**Table 2:** GRIPP2 Short Form Reporting Checklist

GRIPP2-Short Form Item	Description	Page Number
1. Aim: Report the aim of PPI in the study.	The aim of PPI (widely referred to as Consumer and Community Involvement [CCI] in Australia and used hereafter) was to co-develop the Health in Preconception, Pregnancy and Postpartum (HiPPP) Global Alliance’s CCI Framework.	1, 3
	The aim of the co-developed CCI framework is to better enable opportunities for consumer involvement in health research during preconception, pregnancy and postpartum life stages, so as to establish not only what “what works” but also “what translates” into meaningful health outcomes for women and the next generation.	3
	Consumers (widely accepted term in Australia for public contributors and used hereafter) would have central roles in co-designing, co-implementing and co-disseminating the strategy and framework here does not need title case, for consistency with rest of document.	2-3
2. Method: Provide a clear description of methods used for PPI in the study.	The HiPPP CCI Framework CCI team included two consumers (LJ and HiS) from established non-governmental women’s health and consumer representative organisations in Europe, and six consumers (including AE, MH, SJ, PT) recruited via a playgroup in Melbourne, Australia. The recruitment process entailed the provision of written and audio information outlining the project and telephone discussions with HB to allow consumers to ask questions before agreeing to join the team. Consumers in Europe received training through their organisations regarding involvement as consumer experts in research activities. Consumers in Melbourne received training facilitated by HB. Training included consideration of how their unique insights/perspectives would add value to the project.	4
	Consumers were recruited to attend a workshop and refine the values and pragmatic approaches described in the framework. A combination of group and individual face-to-face meetings in a variety of locations in Europe and Australia, emails and telephone communication systems were used to accommodate consumers’ differing availability.	7
	Collaborations were facilitated by HB, in consultation with international CCI experts. All consumers were invited to approve the final version of the framework, and they received feedback on how their input was incorporated, and co-author this manuscript.	9–10
3. Study results: Outcomes – report the results of PPI in the study, including both positive and negative outcomes.	Consumers provided perspectives, based on reflections from their own experiences, on how the values and approaches incorporated in the framework could translate into enabling more meaningful consumer involvement opportunities during preconception, pregnancy and postpartum life stages. This was particularly important for addressing the overall aim of the co-developed framework, which was to address common barriers that might prevent consumers from being involved in research during important maternal obesity prevention life stages and provide realistic actionable solutions for increasing consumer CCI awareness and involvement.	Table 1 Figure 1 Supplemental Table 3
4. Outcomes – comment on the extent to which PPI influenced the study overall. Describe positive and negative effects.	All consumer input was incorporated in the final version of the framework. Involving consumers with current/recent experience of HiPPP’s research focus provided an opportunity to better understand CCI values and “real world” approaches that might be relevant to potential HiPPP consumers, such as settings for reaching consumers, modes of preferred communication methods and ways that involvement could provide value to their lives beyond monetary.	Table 1 Figure 1 Supplemental Table 3
	While the framework incorporates perspectives of consumers from a range of cultural backgrounds, it does not incorporate the diversity of voices represented across all communities.	10-11
5. Reflections/critical perspective: Comment critically on the study, reflecting on the things that went well and those that did not, so others can learn from this perspective.	CCI worked well in the study. However, it may not be feasible for all researchers to provide the flexibility that was offered to conduct meetings at irregular times, variety of child-friendly settings, and feedback in a range of formats. Mutually trusting researcher-consumer relationships are essential for successful CCI and these types of high-quality relationships can take time to nurture. More time and resources are needed to expand the diversity of voices included in the framework.	10-11

*PPI=Public and Patient Involvement, also referred to as CCI=Consumer and Community Involvement; HiPPP=Health in Preconception, Pregnancy and Postpartum*

### Phase 1: the HiPPP CCI workshop

Thirteen international experts in the fields of women’s preconception, pregnancy and postpartum health, two consumers and six early career researchers attended a CCI workshop in September 2018 [14]. Five continents were represented. The consumers (LJ and HiS) were invited from established non-governmental women’s health and consumer representative organisations and had received training through these organisations regarding involvement as consumer experts in research activities. Our aim for the workshop was to establish values and approaches for implementing meaningful CCI as a foundation for the HiPPP CCI Framework. Given previous CCI tokenistic approaches and conceptual challenges identified in the literature [22, 28], we sought to clarify the Alliance’s vision and objectives around meaningful CCI. The workshop discussion was guided by two overarching questions: 1. What do we mean by “partnering” with consumers and community? and 2. How might we ensure that CCI initiatives are meaningful? The discussion was recorded live and discussion points were summarized by HB (Supplemental Table 1). Outputs then informed *Phases 2 and 3* of the development of the HiPPP CCI Framework.

### Phase 2: literature review

Two reviews of CCI frameworks implemented in health and medical research have been recently published [26, 32]. Miller et al.’s [26] review was conducted to inform the co-design of a framework to guide CCI initiatives at an Australian-based research institute. This review highlighted the dearth of evidence-based methodologies for conducting meaningful CCI. While Miller et al’s evidence-based framework had merit, its overall focus was not transferrable to the HiPPP context because it was designed to guide initiatives embedded within a particular research institute and did not address common barriers to CCI in the preconception, pregnancy and postpartum life stages. Similarly, despite identifying 65 CCI frameworks from the literature, Greenhalgh et al.’s review [32] concluded that “off the shelf” frameworks offer limited transferability because each research focus will require adaptations to suit individual relevant contexts. For example, approaches used to involve consumers in obesity prevention research, particularly if consumers are not already engaged in a clinical service, are likely to differ from those outlined in frameworks designed to involve consumers who are recipients of a health service. Hence, we sought to examine the literature for evaluations of CCI frameworks that were specifically designed for use within the obesity research context. Our goal was to highlight frameworks, principles and initiatives that could be used to inform the development of the HiPPP CCI Framework.

#### Peer-reviewed literature

The academic databases MEDLINE Complete and CINAHL Plus were searched using a combination of the following terms: Consumer; community; stakeholder; patient; public; involvement; engagement; participation; maternal obesity; obesity; lifestyle intervention; framework; guidelines; approach. We searched for peer-reviewed articles published in English between September 2008 and September 2018. This search yielded 451 articles of which all titles and abstracts were reviewed by HB and HS. None of the articles focused on outlining CCI frameworks used to implement meaningful obesity initiatives that were specifically relevant to woman in preconception, pregnancy and postpartum. Nevertheless, four papers provided helpful insights on CCI values and approaches used to partner with communities to co-design and implement obesity interventions targeting other populations (e.g. children/adolescents) [34–37]. Each of the four studies used community-based participatory research to involve relevant consumers such as parents (low income) [37] and communities (e.g., Sub-Saharan community members living in Australia [34]; schools and local community partners [35, 36] in the design and implementation of obesity prevention programs and/or interpretation and dissemination of results. While theoretical models guiding intervention targets differed (e.g., cultural competence framework [34], Family-Centered Action Model of Intervention Layout and Implementation [37]), all programs sought consumer/community perspectives from the inception of each project. As highlighted in Davison et al. [37], one program explicitly emphasized that parents were viewed as experts in their experiences of parenting and family context and equal partners with other professionals and researchers working on the program and formed the majority of the decision-making body. Viewing consumers as experts of their lived experience and equitable partners aligned with the CCI values and approaches established at our workshop in *Phase 1,* and was a concept incorporated into the draft of the HiPPP CCI Framework workshopping sessions described in *Phase 3*.

#### Grey literature

We also conducted a targeted search of the websites of five leading national bodies to identify their CCI frameworks, approaches and related resources: the Australian National Health and Medical Research Council Standards for Consumer and Community Involvement [15], the Australian Department of Health Stakeholder Engagement Framework [27], the UK National Institute for Health Research INVOLVE [25], Patient-Centered Outcomes Research Institute in the US (PCORI) [24] and the Canadian Strategy on Patient-Oriented Research (SPOR) [23]. These websites provide information on CCI best practice principles, including recognition, training needs, policies and resources for implementing meaningful CCI. As a recently formed Alliance, HiPPP can benefit from previous efforts to establish CCI best practice. Although the five frameworks noted above were designed for a broad health research focus, they provide transferrable foundational values and approaches on which to build the HiPPP CCI Framework. For example, each framework emphasized the importance of facilitating inclusive CCI to promote equity and relevance, involving consumers at various levels and stages of the research cycle, valuing consumers’ time and expertise, and providing capacity building opportunities for researchers and consumers.

Hence, the overarching CCI values and approaches that we identified in these five frameworks most relevant to HiPPP were captured and are described in Supplemental Tables 2 and 3. Firstly, the identified CCI values and approaches were used to guide *how* we partnered with consumers in *Phase 3*. For example, consumers were involved in decision-making around logistics (i.e., scheduling of meeting times and locations) and workshopping and refining the CCI values and approaches incorporated into the framework [15, 25, 27]. Consumers received verbal and written information detailing how their contributions would add value to the research as well as feedback relating to how their input was used [25]. Training was provided to researchers and consumers to enhance their capacity to partner in research [15, 25]. Secondly, the identified CCI values and approaches informing the development of our proposed CCI framework (earlier versions of Supplemental Tables 2 and 3), were reviewed and refined further during consultations and collaborations outlined in *Phase 3*.

### Phase 3: consultations and collaborations

#### Targeted consumer involvement

Consumer involvement was targeted in Phase 1 as they were key members of the HiPPP Alliance. To ensure CCI in the interpretation of the outcomes of Phase 2, we recruited six consumers (five women and one male partner) via a playgroup based in Melbourne, Australia, word of mouth and professional contacts with Aboriginal women’s health advocates/midwives. One of the consumers was born in Venezuela and spoke English as a second language; her experiences of perinatal care were through the Australian health system. These individuals had recent or current lived experience of healthy lifestyle-related issues, including suboptimal diet quality and physical activity levels and/or excess body weight, during preconception, pregnancy and/or postpartum. Three consumers (one of the women and the couple) had one child under the age of two, one consumer had two children aged 2 and 4 years and another consumer had three primary (elementary) school-aged children. The sixth consumer was in the preconception phase. Two of the women had experienced fertility issues associated with having polycystic ovary syndrome, one developed gestational diabetes during her three pregnancies and another was hospitalised during both of her pregnancies due to Hyperemesis Gravidarum (excessive vomiting and nausea). During initial discussions about the project, most consumers volunteered information about their own experiences of attempts to engage in healthy lifestyle practices, including detailing challenges in maintaining optimal dietary intake, physical activity levels and weight management in preconception pregnancy and postpartum. In addition to noting the relationship between their perinatal eating and weight patterns and current weight status, most consumers stated that they wished they had known earlier that un/healthy lifestyle behaviours before, during and after pregnancy could impact their future child’s health and weight trajectory. As such, consumers viewed HiPPP’s research agenda as a public health priority.

HB met face-to-face with each consumer to workshop the outputs of Phase 2 and to solicit input into the draft of the HiPPP Framework. Consumers were invited to suggest meeting times and locations that would best meet their needs, including making allowances for their family caregiving responsibilities. Meetings were conducted at private residences, a playgroup and cafes during morning or afternoon tea sessions (provided by the research team). On two occasions, meetings were held as “playdates” involving two parents (the researcher and consumer) and their two toddler-aged children at a time, as this format was preferred by some of the consumers who wished to attend with their young children. In these instances, age-appropriate snacks and toys were provided so that consumers could comfortably supervise their children while workshopping the HiPPP CCI Framework.

During the meetings, consumers were invited to have input into co- designing the HiPPP CCI Framework around the results from Phase 1 and 2 activities (Supplemental Tables 1–3). Table 1 provides an overview of the HiPPP CCI values and extends on these values by unpacking barriers and opportunities for implanting meaningful CCI within the HiPPP context. Consumers were also invited to discuss how being involved in research could bring value to their own personal and professional lives, beyond the monetary. Feedback obtained during the meetings was documented by HB and in consultation with consumers, informed the framework.

Workshopping the HiPPP CCI Framework with consumers helped to identify opportunities for implementing meaningful CCI during the specific life stages of preconception, pregnancy and postpartum. The framework includes five key value principles for guiding the implementation of meaningful CCI initiatives. The framework needs to be: 1. Inclusive; 2. Flexible; 3. Transparent; 4. Equitable and 5. Adaptive (Supplemental Table 2). The framework also encompasses pragmatic considerations (Supplemental Table 3) that need to be made to address common barriers that may prevent consumers from participating in and partnering with research throughout the preconception, pregnancy and postpartum periods and provides pragmatic suggestions for engaging them during each stage. For example, given that consumers in preconception may not be aware of opportunities for CCI, the framework suggests potential targeted environments (i.e., the workforce) and methods (i.e., podcast; social media) for promoting involvement. The framework also acknowledges the value of the lived experience that consumers bring to research and translation initiatives by providing examples of remuneration (monetary; vouchers; childcare allowance to attend meetings) and professional opportunities such as co-authoring research papers and co-presenting at conferences (which were of particular interest to women wishing to re-enter the workforce after taking extended caregiving leave).

#### International consultations

Ongoing discussions were held with international experts in research and consumer involvement via virtual and face-to-face (in Australia and the US) meetings to further define processes and principles to support and enhance meaningful consumer involvement. Additionally, one expert from Canada (author VVW) and another from the UK (author MS) were invited to Monash University, Australia to provide intensive CCI training and education to research team members as well as offer additional international insights based on years of experience implementing CCI. Applicable insights and information were incorporated during the workshopping of the framework as summarised in Table 1.

Consultations with CCI experts highlighted the need to think ‘outside of the box’ to develop time-efficient, relevant and accessible CCI opportunities. For example, in the UK, CCI researchers organised free ‘Yoga for Bump’ (antenatal yoga) sessions, held in a disadvantaged locale, to engage women in maternity-related discussions with a researcher and Q&As with a midwife [38, 39]. The main aim of the initiative was to create more accessible opportunities for consumers from a range of backgrounds to be involved in research. The initiative also sought to address competing time demands that often prevent pregnant women from being involved in research by organising an activity that they might enjoy taking part in and instead of expecting women to travel to the research institution, they held sessions in a more convenient location. Yoga for Bump Sessions were promoted through flyers, posters, social media and the group website [39].

Discussions with CCI experts also addressed potential power differentials between researchers and consumers as well as approaches for nurturing relationships and therefore equitable partnerships. An example of an activity used to promote equitable partnerships involved matching a researcher and consumer during a lunch, held directly before the start of project meetings. Matched researcher-consumer pairs were assigned the task of learning about each other over their meal and introducing one another to the group at the meeting. CCI experts also highlighted the importance of avoiding making assumptions about consumers’ knowledge, interest and ability to learn about technical aspects of health research and the need for researcher-consumer partnerships to establish a shared understanding of what is needed from both sides.

## Discussion

The aim of this paper was to outline the processes involved in the development of the CCI framework for the Global HiPPP Alliance, formed to improve healthy lifestyle in reproductive aged women, prevent obesity and optimise health of women and the next generation globally. Our strategy involved three phases, encompassing: 1. An initial workshop with experts, including consumers, researchers and clinicians to establish our values and approaches for conducting meaningful CCI; 2. A review of CCI frameworks and grey literature; and 3. Collaborations with consumers and CCI experts to further refine the framework.

Our collective efforts across all three phases highlight the advances that have occurred over the past years to expand CCI through investment in research and the development of guidelines and frameworks [22, 30, 32, 40]. This work has enabled us to explore integral organisational [41], researcher and consumer level [14, 25] factors that need to be considered when developing the CCI framework. Yet, lessons learned during the developmental phase of our CCI framework supported other research findings showing that underpinning CCI values are rarely made explicit [42] and there is a paucity of published research and resources that describe pragmatic steps for implementing researcher and consumer engagement during different stages of preconception, pregnancy and postpartum [13]. The findings from Greenhalgh et al.’s [32] systematic review of generic CCI frameworks indicated that existing frameworks provided limited transferability and that it may be beneficial to use a number of evidence-based resources alongside stakeholder involvement for co-designing targeted frameworks. Similarly, our work describes how combined efforts from researchers, CCI experts and consumers can be valuable in shaping a framework for HiPPP’s specific areas of focus. Indeed the values adopted by our framework aligned well with values that informed the development of the Alberta SPOR Unit Patient (in Canada the term patient refers to a person/consumer with lived experience of a health issue or their informal caregiver, such as a relative or friend) Engagement Platform [23], which were shared with us by one of the international CCI experts (VVW) on the team (e.g., Guidelines on Compensation [43]; Ethical Guidelines for Engaging with Patients as Researchers [44]). Additionally, our work to date supports the findings from a recent review of progress in CCI in the UK and internationally [22], which highlighted an increased need for the reporting of and evaluation of CCI components of research and translation initiatives. It is likely that increased efforts to report on all aspects of CCI, including the development of frameworks, will provide the conceptual as well as practical knowledge needed to drive broader implementation of meaningful researcher and consumer involvement [42].

Despite the lack of published research incorporating CCI and resources for the specific stages of preconception, pregnancy and postpartum, we benefited greatly by learning from generic CCI research and best practices. This includes translating ‘what works’ from differing contexts than those of our framework, and undoubtedly, we will continue to evolve and adapt it as we increase our understanding about ‘what works’ in the context of HiPPP. In particular, we are mindful of barriers preventing consumers from being involved in research during the preconception, pregnancy and postpartum periods and, as such, our framework focuses on providing realistic and practical solutions to address these barriers, including considerations for potential constraints associated with being a family caregiver. However, we envisage that these too will be adapted in response to the voices of consumers with experiences from varied backgrounds and contexts, including consumers from Indigenous and culturally and linguistically diverse backgrounds. For example, an intervention designed for women in the general population who are engaged with perinatal health services may not be as relevant to women from vulnerable populations or those living in remote communities. This requires CCI to be undertaken with a deep understanding of historical, cultural and social complexity of specific local or regional contexts [45]; values that benefit all. Building the mutually respectful relationships needed to carry forward this work will take time.

Furthermore, we have discussed preconception, pregnancy and postpartum life stages from a broad perspective, however the framework may be applicable to other research areas that are relevant to these life stages, such as Polycystic Ovary Syndrome, Gestational Diabetes and assisted reproductive technology. We also note that our peer reviewed literature search may not have captured all relevant studies as a wide range of terms are used across the CCI literature [16]. Furthermore, our grey literature search was limited to the peak bodies that had emerged during discussions with researchers and other experts working in the field at the time, meaning that other valuable resources may have been omitted. Indeed, following the co-development of the framework we have become aware of women’s health research and services that are likely to implement innovative methods for involving consumers in research and co-production activities, but even then have not been able to identify accessible resources outlining the pragmatic approaches implemented to facilitate meaningful CCI that extend beyond the research participant level.

Finally, research evidence [46] suggests that to date, there is not any one particular method of choice to support effective CCI [16, 46]; hence there may be a range of other approaches, including how consumers are recruited (e.g., via professional networks; advertising) that may be just as useful, and we would emphasize that the overarching project’s goals, resources, and who may be the best people for the project will be key in guiding those decisions. Indeed, our ongoing consultations with international experts fast-tracked our in-depth learning of conceptual and pragmatic aspects of CCI (including systems and tools) that would have been difficult to attain through CCI literature or related resources alone. The tacit knowledge and guidance the international experts have imparted during the development of the framework reflects the vantage point they bring based on years of working with researchers and consumers to solve health problems in the real world. Many of the perspectives relating to partnering with consumers that have helped to shape our framework development and processes – including those around power differentials, CCI recruitment approaches and dissemination initiatives – have resulted from this engagement with them. This emphasizes that in the absence of comprehensive CCI reporting, collaborations with others with vast knowledge and experience in this area are essential. With regards to collaborating with consumers, many of the consumers expressed that being offered accessible (i.e., flexible times; child-friendly) meeting arrangements, feedback on how their input was being incorporated into the framework, and invitations to co-author the current article helped them to better understand the value that they brought to the project as well as feel more connected with HiPPP’s broader aims. As HiPPP’s agenda expands, we will invite both researchers and consumers to provide reflections on both positive and negative aspects of their involvement. Lessons learned will be incorporated into the framework as it evolves.

## Future directions

The Global HiPPP Alliance is an international network. In 2018 we workshopped the development of a CCI framework with international stakeholders including consumers. Post-workshop activities (Phases 2 and 3) were based on the literature, experiences of Australian consumers and international CCI experts. It is acknowledged that further international consumer involvement is needed. Our strategy acknowledges the importance of local and cultural context and we have held preliminary discussions with women from Aboriginal and Torres Strait Islander communities in Australia. We will continue to work on adapting the framework as these partnerships and others develop. The framework has the capacity to be adapted to local settings (e.g., the clinical setting may not be relevant across all contexts, however the framework provides scope for including more culturally relevant terms and locations) and the next step is to include new learnings from Australia as well as the work from our international collaborators. Importantly, we recognise that those with whom we partner with in research will vary according to local cultural contexts as will the methods used for researcher and consumer involvement.

## Conclusion

Frameworks for researcher and consumer involvement during the preconception, pregnancy and postpartum periods must consider barriers and opportunities associated with these specific life stages. We have drawn on comprehensive literature searches and involvement at all stages with CCI experts and consumers to co-develop a CCI framework that is relevant to HiPPP consumers and can be adapted to local contexts and evolving needs of research and implementation initiatives. Implementation and evaluation of the HiPPP CCI Framework is needed to understand its effectiveness in facilitating meaningful partnerships between researchers and consumers that result in improved healthy lifestyle outcomes for women in preconception, pregnancy and the postpartum.

## Supplementary information

**Additional file 1: Table 1.** Summary of the HiPPP CCI Framework workshop discussion points

**Additional file 2: Table 2.** The HiPPP CCI Framework values and approaches summarised.

**Additional file 3: Table 3.** Pragmatic approaches for facilitating meaningful CCI in preconception, pregnancy and postpartum for improving healthy lifestyle and reducing maternal obesity.

## Data Availability

Documentation and materials relating to phases 1–3 are summarised within the manuscript.

## Abbreviations

CCI: Consumer and community involvement
HiPPP: Health In Preconception, Pregnancy and Postpartum
