# Supplementary material for: Global Health in Preconception, Pregnancy and Postpartum Alliance: development of an international consumer and community involvement framework
Source: Res Involv Engagem. 2020 Aug 10;6:47. doi: 10.1186/s40900-020-00218-1 (PMC7419190; doi:10.1186/s40900-020-00218-1)
Supplement: Supplementary file 1 — Additional file 1: Table 1. Summary of the HiPPP CCI Framework workshop discussion points [file 40900_2020_218_MOESM1_ESM.docx]

**Table 1.**

*Summary of the HiPPP CCI framework workshop discussion points*

| ***Values***   - *Inclusivity:* All research and translation initiatives need to be inclusive of CCI throughout all stages from early conception to dissemination. - *Respect:* Mutual respect for all perspectives, beliefs, values, culture and regional context, whilst also acknowledging differences - *Shared purpose:* Seeking alignment of purpose across stakeholders is important. - *Valuing contribution:* Time and effort should be valued and respected by all. - *Consideration:* Language, terminology or ‘labels’ are important and need careful consideration and agreement - *Open contribution:* Consumers should be ensured they have opportunities (i.e., feel confident and comfortable) to contribute. |
| --- |
| ***Approaches*** |
| - Deploy and engage existing and new partners and relationships to build collaboration and support in this area. - Reach international/regional trusted contacts/leaders. - Identify broad and relevant stakeholders including those with lived experiences. - Consider broad stakeholders, not just in terms of outcomes (e.g., in our case, it may be anyone who feels connected to the issue, such as partners, not just women during preconception or pregnancy stages). - Existing groups with lived experience need to be captured. - A multifaceted strategy is needed to capture a broad demographic spread; for each purpose a different strategy may be needed. - Consider language and terminology (i.e., women or families, weight or health etc); this can be advised by CCI members. - Ensure cultural relevance and sensitivity. - Rules of engagement, clear governance and transparency needed in all stages. - Training should be provided, including to show consumers how their experience holds value - Peer leaders to be educated to educate others in range of settings and feedback. - Consider forming an international networking CCI group. |
| ***Additional points*** |
| - Need to stress that reach and relevance is broader than the included women - Consider healthy lifestyle and health outcomes, not just weight |
