# Supplementary material for: Global Health in Preconception, Pregnancy and Postpartum Alliance: development of an international consumer and community involvement framework
Source: Res Involv Engagem. 2020 Aug 10;6:47. doi: 10.1186/s40900-020-00218-1 (PMC7419190; doi:10.1186/s40900-020-00218-1)
Supplement: Supplementary file 2 — Additional file 2: Table 2. The HiPPP CCI Framework values and approaches summarised. [file 40900_2020_218_MOESM2_ESM.docx]

**The HiPPP CCI Framework Values and Approaches Summarised**

| **Inclusive** |
| --- |
| - HiPPP works with consumers to understand what is needed to ensure that consumer involvement opportunities are inclusive for women with lived experience of weight/healthy lifestyle issues, regardless of their background, education, location, age, culture and language. This includes holding meetings in appropriate venues (i.e., child friendly), providing interpreters, welcoming support people (i.e., partner) and adapting processes for cultural relevance. - HiPPP has mechanisms and processes to ensure that consumers are compensated for their involvement. This may include reimbursement of transport and childcare costs. - We will produce Plain English Summaries of research available for consumers and local communities. |
| **Flexible** |
| - HiPPP partners with consumers and communities to identify different ways they can be involved in research projects at different stages of the research cycle, that take into account flexible options required to facilitate meaningful involvement during preconception, pregnancy and postpartum. - Decisions on locations, frequency, timing of meetings are made with consumers and local communities. This includes making sure that opportunities for involvement are held at times and places that best suit women at different stages of preconception, pregnancy and postpartum. - HiPPP uses a variety of methods to involve consumers and local communities targeted at specific stages of preconception, pregnancy and postpartum or as a continuum. This includes a CCI section on HiPPP webpages, social media platforms (e.g. Facebook, Whatsapp) and promotion through poster/leaflet campaigns at playgroups, workforces and hospitals. |
| **Transparent** |
| - Opportunities to be involved in research projects in HiPPP will be presented in a clear, accessible format and will include information about expectations for the role, expected time commitment, information about reimbursement and contact details for the lead researcher. - HiPPP will advertise opportunities for involvement in a range of places, including: community centres, hospitals, support groups, playgroups and the workforce. - When opportunities for involvement are advertised, HiPPP will be clear about who they want to recruit and why people have been chosen to be involved (e.g. relevant lived experience) |
| **Equitable** |
| - HiPPP views researcher-consumer relationships based on mutual trust, integrity and respect as central to implementing meaningful CCI - HiPPP recognises the value that both the researcher and consumer contribute toward research - HiPPP consumers are involved in designing and agreeing on CCI strategies - HiPPP researchers working with consumers have the skills to facilitate appropriate group processes, such as fostering a culture that views researchers and consumers as equal partners, creates opportunities for speaking and listening and welcomes diverse viewpoints. - HiPPP partners with consumers to identify ways to publicly acknowledge the value of their contribution, such as co-authoring publications and co-presenting at conferences. - HiPPP recognises that both consumers and researchers may require training and support for implementing CCI - We avoid making assumptions about consumers knowledge and capacity to learn, and partner with them to understand their preferences around the use of medical terminology. |
| **Adaptable** |
| - HiPPP evaluates researcher and consumer experiences and involvement at set intervals throughout the project and adapts processes as the needs of consumers, researchers and HiPPP change. - HiPPP will nurture a culture where consumers feel comfortable providing feedback about their experiences and discuss altering their roles at any stage. - HiPPP ensures that consumers have a clear idea of how their involvement contributes toward the research and its outcomes - We develop methods to evaluate the impact of CCI on improving healthy lifestyle of women in preconception, pregnancy and postpartum. - Researchers provide regular feedback to consumers and communities on their involvement in projects and in turn receive feedback on consumers’ reflections on experiences of involvement, making changes to involvement approaches where necessary. |
