# Supplementary material for: Global Health in Preconception, Pregnancy and Postpartum Alliance: development of an international consumer and community involvement framework
Source: Res Involv Engagem. 2020 Aug 10;6:47. doi: 10.1186/s40900-020-00218-1 (PMC7419190; doi:10.1186/s40900-020-00218-1)
Supplement: Supplementary file 3 — Additional file 3: Table 3. Pragmatic approaches for facilitating meaningful CCI in preconception, pregnancy and postpartum for improving healthy lifestyle and reducing maternal obesity. [file 40900_2020_218_MOESM3_ESM.docx]

**Table 3: Pragmatic approaches for facilitating meaningful CCI in preconception, pregnancy and postpartum for improving healthy lifestyle and reducing maternal obesity.**

| **Preconception** | **Pregnancy** | **Postpartum** |
| --- | --- | --- |
| **Where we involve women with lived experience^1^: Settings and methods of reach** | | |
| Primary care (GP, community health) | Primary care (GP, community health) | Primary care (GP, community health) |
| Hospitals (e.g., before discharge in postpartum- inter-conception) | Pregnancy care / Hospitals | Postpartum follow up – Hospitals postpartum visit |
| Specialist care (Obgyn, assisted reproductive technology services; endocrinologists, others) | Specialists and other care providers (e.g. Obgyn, midwives; doulas (employed birthing support person who does not typically have formal training), mental health provides and others) | Specialists and other care providers |
|  |  | Maternal Child Health Nurse |
| Waiting rooms (medical, infertility care; allied health, natural medicine; family planning clinic; primary care). | Waiting rooms (e.g., primary care; obgyn; others) | Waiting rooms (e.g., primary care; others) |
| Workplace | Workplace | Workplace |
| Community Centres (e.g., Migrant Women’s health centres) | Community Centres (e.g Migrant Women’s health centres) | Community Centres (e.g., Migrant Women’s health centres) |
| Library | Library | Toy/book libraries |
|  |  | Playgroup including cultural/ language group |
|  |  | Childcare |
| Targeted health workforce (e.g., Indigenous community worker) | Targeted health workforce (e.g., Indigenous community worker) | Targeted health workforce (e.g., Indigenous community worker) |
| Patients/consumer support groups (e.g., infertility, PCOS) | Patients/consumer support groups (e.g. pregnancy, PCOS, diabetes) | Patients/consumer support groups (e.g., diabetes, PCOS) |
|  |  | Breastfeeding Associations |
| Not for profit organisations (e.g., Diabetes Associations, Women’s Health organisations) | Not for profit organisations (e.g., Diabetes Associations, Women’s Health organisations) | Not for profit organisations (e.g., Diabetes Associations, Women’s Health organisations) |
|  |  | High risk baby support programs (e.g., premature; NICU babies) |
|  |  | Targeted postpartum Mental health support |
| Via social media: organic and paid including targeted groups (e.g., trying to conceive groups). | Via social media: organic and paid including targeted groups (parent/pregnancy and such). | Via social Media: organic and paid; online groups (parent and such). |
| Via media: TV, radio, print, community media | Via media: TV, radio, print, community media | Via Media: TV, radio, print, community media |
| Events (e.g Council and women’s festivals) | Events (e.g Council and women’s festivals) | Events (e.g., Council and those targeting women, families) |
|  | Targeted baby events | Targeted baby events |
| Physical activity venues/ gyms | Physical activity venues/ gyms | Physical activity venues/ gyms |
| Targeted podcasts | Targeted podcasts | Targeted podcasts |
| Retail outlets (e.g., Supermarkets; charity shops) | Retail outlets (e.g., Supermarkets; charity shops) | Retail outlets (e.g., Supermarkets; charity shops) |
| Via CCI database | Via CCI database | Via CCI database |
| Targeted mobile apps | Targeted mobile apps including pregnancy | Targeted mobile apps including on babies |
| Pharmacy: sections for tests (ovulation/pregnancy) and vitamin and minerals. | Pharmacy | Pharmacy |
|  |  | Parenting playgroups |
|  |  | Parent rooms in shopping centres, workplaces |
|  | Support services/programs for young mums | Support services/programs for young mums |
| **How we facilitate opportunities for meaningful involvement^2^** | | |
| Online, telephone and face-to-face | Online, telephone and face-to-face | Online, telephone and face-to-face |
| Reimburse for travel/ childcare costs (inter-pregnancy) | Reimburse for travel/ childcare costs (inter-pregnancy) | Reimburse for travel/ childcare costs |
| Support friend/relative welcome | Support friend/relative welcome | Support friend/relative welcome |
| Interpreter/translation welcome/provided | Interpreter/translation welcome/provided | Interpreter/translation welcome/provided |
| Meet in child-friendly venues (i.e., play groups; child activities where parents are sitting around waiting) | Meet in child-friendly venues (i.e., play groups; child activities where parents are sitting around waiting) | Meet in child-friendly venues (i.e., play groups; child activities where parents are sitting around waiting) |
| Provide afternoon tea or equivalent | Provide afternoon tea or equivalent | Provide afternoon tea or equivalent |
| Preconception starter kit with evidence-based advice and source of information clearly identified and referenced (other online and advice not always accurate/consistent) | Pregnancy information pack with evidence-based advice and source of information clearly identified and referenced (other online and advice not always accurate/consistent) | Baby starter kit with evidence-based advice and source of information clearly identified and referenced (other online and advice not always accurate/consistent) |
| Vouchers for education sessions (e.g., consultation with pre-pregnancy dietitian)/information sessions | Vouchers for education sessions (e.g., consultation with pregnancy dietitian, information sessions) | Vouchers for education sessions (e.g., consultation with post-partum dietitian; child feeding etc) information sessions |
| Invitations to co-author and co-present research and other opportunities that will help boost professional development/CV | Invitations to co-author and co-present research and other opportunities that will help boost professional development/CV | Invitations to co-author and co-present research and other opportunities that will help boost professional development/CV |
| Provide certificates to recognise training received or involvement | Provide certificates to recognise training received or involvement | Provide certificates to recognise training received or involvement |
| **Communication^3^** | | |
| Provide options for low literacy levels | Provide options for low literacy levels | Provide options for low literacy levels |
| Basic English as a general rule but ask consumers what they would like (don’t make assumptions). | Basic English as a general rule but ask consumers what they would like (don’t make assumptions). | Basic English as a general rule but ask consumers what they would like (don’t make assumptions). |
| Antenatal packs (with standout item) | Antenatal packs (with standout item) |  |
|  |  | Maternal Child Health Nurse information packs |
| Short brief messages | Short brief messages | Short brief messages |
| Posters | Posters | Posters |
| Social media posts and videos | Social media posts and videos | Social media posts and videos |
| Provide health updates and tips | Provide health updates and tips | Provide health updates and tips |
| Informative talks to families (mothers and fathers) | Informative talks to families (mothers and fathers) | Informative talks to families (mothers and fathers) |

^1^The *where we involve women with lived experience* sections highlights settings and methods for promoting CCI opportunities for women in preconception, pregnancy and postpartum as they don’t typically view themselves as patients. ^2^ The *how we facilitate opportunities for meaningful involvement* sections provides examples of approaches for implementing CCI with women in preconception, pregnancy and postpartum that account for their limited availability due to caregiving responsibilities and other barriers to participation (i.e., language; location; disability; culture) and to recognize the value of the expertise that they contribute. ^3^ The *communication* sections outline examples of approaches for communication with consumers in preconception, pregnancy and postpartum that are inclusive and accessible and type of content that provides value to those involved.
